# Asymptomatic COVID-19 Individuals Tend to Establish Relatively Balanced Innate and Adaptive Immune Responses

**DOI:** 10.3390/pathogens10091105

**Published:** 2021-08-30

**Authors:** Miao Li, Yue Zhang, Jianhua Lu, Li Li, Huixia Gao, Cuiqing Ma, Erhei Dai, Lin Wei

**Affiliations:** 1 Department of Immunology, Key Laboratory of Immune Mechanism and Intervention on Serious Disease in Hebei Province, Hebei Medical University, Shijiazhuang 050000, China; 18200739@hebmu.edu.cn (M.L.); 20181014@stu.hebmu.edu.cn (Y.Z.); macuiqing@hebmu.edu.cn (C.M.); 2 The Fifth Hospital of Shijiazhuang, Hebei Medical University, Shijiazhuang 050000, China; 13323219965@163.com (J.L.); 20200268@stu.hebmu.edu.cn (L.L.); 13313215651@163.com (H.G.)

**Keywords:** asymptomatic, COVID-19, SARS-CoV-2, immune responses

## Abstract

The sharp increase in the proportion of asymptomatic cases and the potential risk of virus transmission have greatly increased the difficulty of controlling the COVID-19 pandemic. The individual immune response is closely associated with clinical outcomes and pathogenic mechanisms of COVID-19. However, the clinical characteristics and immunophenotyping features of immune cells of asymptomatic individuals remain somewhat mysterious. To better understand and predict the disease state and progress, we performed a comprehensive analysis of clinical data, laboratory indexes and immunophenotyping features in 41 patients with SARS-CoV-2 (including 24 asymptomatic cases and 17 symptomatic individuals). Firstly, from the perspective of demographic characteristics, the rate of asymptomatic infection was significantly higher in those with younger age. Secondly, the laboratory test results showed that some indexes, such as CRP (acute phase reaction protein), D-Dimer and fibrinogen (the marker for coagulation) were lower in the asymptomatic group. Finally, symptomatic individuals were prone to establishing a non-protective immune phenotype by abnormally decreasing the lymphocyte count and percentage, abnormally increasing the Th17 percentage and decreasing Treg percentage, which therefore cause an increase in the neutrophil/lymphocyte ratio (NLR), monocytes/lymphocytes ratio (MLR) and Th17/Treg ratio. On the other hand, asymptomatic individuals tended to establish a more effective and protective immune phenotype by maintaining a normal level of lymphocyte count and percentage and a high level of NK cells. At the same time, asymptomatic individuals can establish a relatively balanced immune response through maintaining a low level of monocytes, a relatively low level of Th17 and high level of Treg, which therefore lead to a decrease in MNKR and Th17/Treg ratio and finally the avoidance of excessive inflammatory responses. This may be one of the reasons for their asymptomatic states. This study is helpful to reveal the immunological characteristics of asymptomatic individuals, understand immune pathogenesis of COVID-19 and predict clinical outcomes more precisely. However, owing to small sample sizes, a future study with larger sample size is still warranted.

## 1. Introduction

In December 2019, the coronavirus disease 2019 (COVID-19), caused by severe acute respiratory syndrome coronavirus 2 (SARS-CoV-2) infection, broke out in China. As of 18 August 2021, the WHO reported that there had been 208,470,375 confirmed cases, including 4,377,979 deaths globally. So far, there are no specific antiviral treatments. According to Diagnosis and Treatment Protocol for COVID-19 Patients (Tentative 8th Edition) of the National Health Commission of the People’s Republic of China, confirmed cases could be classified into four clinical types: mild cases, moderate cases, severe cases and critical cases [1]. In the early stage of the outbreak, most medical resources tended to identify and treat critical cases. With the discovery of the risk posed by asymptomatic transmission, it is of great significance to pay more attention to asymptomatic individuals to contain outbreaks.

Asymptomatic infected people refer to those whose respiratory tract specimens are positive for the nucleic acid test of SARS-CoV-2 but do not show any relevant clinical symptoms, such as fever, cough, sore throat and other self-perceived or clinically recognizable symptoms. Recently, many studies showed that there might be an increase in the asymptomatic proportion of SARS-CoV-2 globally. According to the early epidemiology report from the Chinese Center for Disease Control and Prevention (China CDC), there were 889 asymptomatic COVID-19 cases among 44,672 confirmed cases, accounting for about 2.0% of the total confirmed cases [2]. The latest retrospective cohort study conducted by China CDC assessed the proportion of asymptomatic infection in all international entrants from 16 April to 12 October 2020. The results showed that among 3130 SARS-CoV-2 positive entrants, 1612 (51.9%) were considered to have asymptomatic infection. Furthermore, the proportion of asymptomatic infections increased significantly from 27.8% (from 16 April to 30 April) to 59.4% (from 28 September to 12 October 2020) [3]. Additionally, Iceland reported that 43% of the participants positive for the nucleic acid test had no symptoms based on a large-scale population test, which can accurately reflect the real situation of the entire population [4]. All this research implied a sharp increase in asymptomatic infection at a global scale. More and more evidence showed that asymptomatic individuals could greatly accelerate the spread of the virus from person to person, which caused great difficulties in the control of COVID-19 [5,6,7]. Therefore, early screening, early reporting, and early separation of asymptomatic individuals may be a key breakthrough to control the COVID-19 pandemic [8].

As we know, the clinical outcome of viral infection is closely associated with the virus itself, the amount of virus, the manner of infection, and the characteristics of the host immune response. However, our understanding of the clinical characteristics and immunophenotyping features of asymptomatic COVID-19 individuals remains somewhat mysterious. The occurrence, clinical outcome and screening of treatment targets of COVID-19 are all closely related to the host immunity. It is critically important and urgent to fully understand the immune mechanism of asymptomatic state, which will be beneficial to predict the outcome of the disease through dynamic observation of immune phenotype. Therefore, we can intervene and improve the prognosis of patients as soon as possible. Besides, the revelation of immunophenotyping characteristics will be helpful to provide a new target of immunological intervention. Overall, immunological findings of asymptomatic patients are crucial in the fight against SARS-CoV-2. This study collected 41 COVID-19 patients (including 24 asymptomatic cases and 17 symptomatic cases with moderate or severe clinical manifestation) admitted to the Fifth Hospital of Shijiazhuang from January to June 2020.

On the one hand, we carried out routine laboratory tests. On the other hand, we collected the peripheral blood before any clinical intervention and monitored the number and proportion of various immune cells and lymphocyte subsets. This study aims to explore the immunological characteristics of asymptomatic cases, understand the immune pathogenesis of COVID-19 and predict clinical outcomes more precisely.

## 2. Results

### 2.1. Demographic Characteristics

We collected a total of 41 COVID-19 patients admitted to the Fifth Hospital of Shijiazhuang from January to June 2020, including 24 asymptomatic cases and 17 symptomatic cases (15 moderate cases and 2 severe cases). Asymptomatic infected people included in this study are defined as those with a positive result for the nucleic acid test but without any self-perceived or clinically recognizable symptoms in the preceding 14 days. The demographic characteristics of those two groups are shown in Table 1.

Asymptomatic individuals (24 cases, M/F 10/14, mean age 28 years, range 17–51 years, mean ± S.D. 27.4 ± 9.6 years) and symptomatic patients (17 patients, M/F 11/6, mean age 44 years, range 20–67 years, mean ± S.D. 44.9 ± 18.4 years) had different demographic characteristics. Symptomatic cases were older than asymptomatic individuals (Figure S1). Overall, the percentage of symptomatic and asymptomatic infection in our study was 41.4% (17/41) and 58.5% (24/41), respectively. To further clarify whether age is a possible factor affecting the outcome of infection, we divided the patients into two groups according to age: the older group (older than 40 years old) and the younger group (younger than 40 years old). The incidence of asymptomatic and symptomatic infection was analyzed. It was found that there were significant differences in the rates of asymptomatic and symptomatic infection between those two groups (Table 2). The rate of asymptomatic infection was higher in the younger group, whereas the rate of symptomatic infection was higher in the older group.

Furthermore, we analyzed the influence of gender or smoking on the outcome of infection. As a result, neither gender nor smoking significantly affected the asymptomatic or symptomatic infection (Table 3 and Table 4). Additionally, there was no significant difference in asymptomatic and symptomatic infection rates between local and overseas cases (Table 5).

It is worth noting that more studies are needed to confirm the above results because of the limited sample size. This limitation applies to the following results, and we will not repeat them.

### 2.2. Clinical Characteristics

Baseline characteristics of the patients with COVID-19 in different groups are shown in Table 6. Among these, we focused on and analyzed the following aspects.

Firstly, we analyzed the effect of chronic diseases (including hypertension, cardiovascular disease, respiratory disease and tumor) on the rate of asymptomatic and symptomatic infection and found that chronic diseases exhibited great effect (Table 7). Secondly, we analyzed the effect of the different clinical outcomes on the average hospitalization days and found that average hospitalization days of asymptomatic individuals (16.8 ± 5.2 days) were shorter than that of symptomatic patients (21.9 ± 8.4 days) (Figure S1). After therapies, all the cases were discharged, and none died. Then, the patients were followed up for 4 weeks and were asked to test the nucleic acid once a week. Re-positive was defined as those with a positive nucleic acid test result following four weeks of quarantine. We compared the re-positive rate and found that the re-positive rate of those two groups was equivalent (Table 8).

### 2.3. The Characteristics of Blood Routine Examination, Routine Coagulation Tests and Acute Phase Reactants for the Asymptomatic COVID-19 Individuals

As shown in Figure S2, the main change in blood routine in symptomatic cases was the decrease in absolute number and the percentage of lymphocytes, which were lower than that in the healthy control and asymptomatic group. These changes led to an increase in neutrophil to lymphocyte ratio (NLR) and monocyte to lymphocyte ratio (MLR) in the symptomatic group. In contrast, the main changes in blood routine in the asymptomatic infection group were the increase in neutrophil count and percentage, and the decrease in monocyte count and percentage, which were significantly higher or lower than those of healthy controls, respectively. The results implied that the asymptomatic cases tend top maintain a more favorable immune response by maintaining normal lymphocytes and reducing monocytes to inhibit monocyte-mediated inflammation, which may be helpful to maintain asymptomatic state.

Additionally, we observed blood coagulation indexes in the asymptomatic and symptomatic COVID-19 patients. Among six indexes, the level of fibrinogen and D-Dimer of the symptomatic individuals was significantly higher than that of asymptomatic patients (Figure S3).

As acute reactants, D-Dimer and fibrinogen can act as the coagulation marker and as inflammatory mediators to regulate the inflammatory response [9,10]. Our results reflected that the systemic inflammatory response was relatively strong in symptomatic individuals, which was supported by the differences in the level of CRP. The level of CRP, which is another important indicator of inflammation, far exceeded the normal upper limit in symptomatic cases, while it was in the normal range in asymptomatic individuals (Figure S3).

### 2.4. Immunophenotyping of Lymphocytes, NK and Monocytes

**Figure 1:**
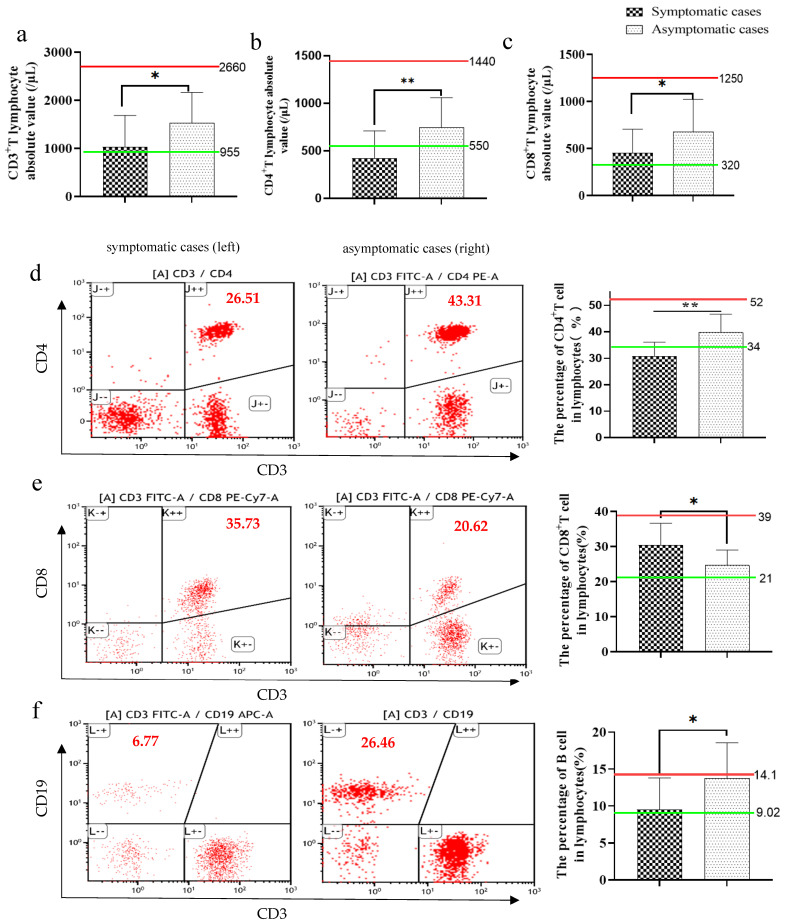
The absolute value and the percentage of CD4 T, CD8 T and B lymphocytes in flow cytometric analysis (gating strategy is shown in supplementary Figure S4) of asymptomatic and symptomatic patients with COVID-19. A representative flow cytometry figure is shown in (**d**–**f**). The following indexes were analyzed, including CD3 T lymphocyte absolute value (**a**), CD4 T lymphocyte absolute value (**b**), CD8 T lymphocyte absolute value (**c**), the percentage of CD4 T cells (**d**), the percentage of CD8 T cells (**e**) and the percentage of B cells (**f**). Green line: the normal lower limit. Red line: the normal upper limit. Each red dot represents single cell. The red numbers in the figure caption represent the percentage of cells in the corresponding quadrants. Bar graphs show the mean ± s.d. * *p* < 0.05; ** *p* < 0.01.

Symptomatic cases had an abnormally low absolute value of CD3^+^ T lymphocyte and their subsets CD4^+^ and CD8^+^ T lymphocyte, which were near or lower than the lower limit of normal range. In contrast, asymptomatic cases kept them in the normal range (Figure 1a–c). Furthermore, we evaluate the changes in the percentage of CD4^+^ T lymphocytes, CD8^+^ T lymphocytes, B lymphocytes and NK cells in the peripheral blood lymphocytes.

**Figure 2:**
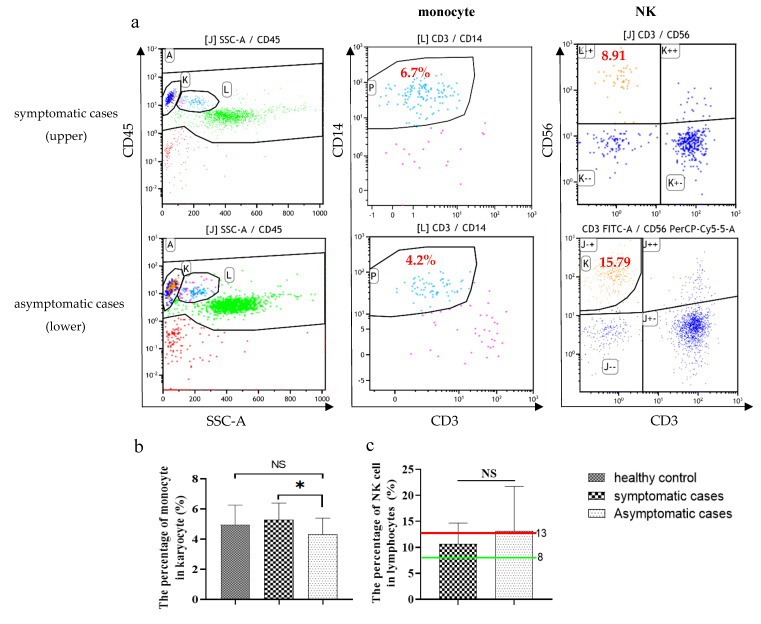
The percentage of NK and monocytes in flow cytometric analysis (gating strategy is shown in supplementary Figure S4) of asymptomatic and symptomatic patients with COVID-19. A representative flow cytometry figure is shown in (**a**). Compared with symptomatic cases and healthy control or normal range, asymptomatic cases had a lower percentage of monocytes (**b**) and a higher percentage of NK cells (**c**). Green line: the normal lower limit. Red line: the normal upper limit. Each dot represents single cell. The red numbers in the figure caption represent the percentage of cells in the corresponding quadrants. Bar graphs show the mean ± s.d. * *p* < 0.05. NS: no statistical significance.

Symptomatic individuals showed an abnormal decrease in a variety of lymphocyte percentages, including CD4 T cell and B cell percentage (Figure 1d,f), while they showed a normal percentage of CD8T (Figure 1e) and NK cells (Figure 2a,c). Those results suggested that the symptomatic group may develop a relatively detrimental immune phenotype. In contrast, the main changes in asymptomatic cases were the increased percentage of B cells (near the upper limit of normal value) (Figure 1f) and the percentage of NK cells (exceed the upper limit of normal value) (Figure 2a,c), and a normal percentage of CD4 T cells and CD8T cells (Figure 1d,e), which demonstrate a relatively effective immune response to protect the host.

In addition, we observed the change in the percentage of monocyte in karyocyte in those two groups based on the baseline of healthy control. We found that the percentage of monocytes was lower in asymptomatic individuals than in healthy control and symptomatic cases. However, the difference between asymptomatic individuals and healthy control did not have statistical significance (Figure 2a,b). This is consistent with the results of blood routine. The low level of monocytes may be beneficial for asymptomatic cases not to produce excessive inflammatory responses, which may be one of the reasons for their asymptomatic state.

### 2.5. Immunophenotyping of some Subsets of CD4^+^ T Lymphocytes

**Figure 3:**
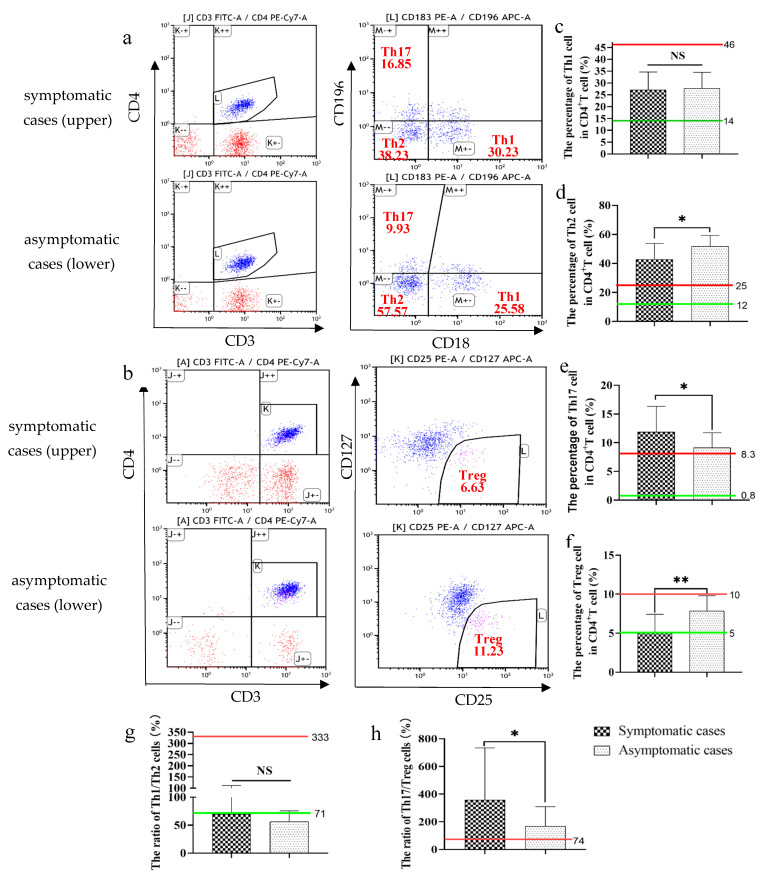
The percentage of subsets of CD4 T lymphocytes in the asymptomatic and symptomatic cases with COVID-19. A representative flow cytometry figure is shown in (**a**,**b**). The following indexes were analyzed, including the percentage of Th1 cells (**c**), the percentage of Th2 cells (**d**), the percentage of Th17 cells (**e**) and the percentage of Treg cells (**f**), the ratio of Th1 to Th2 (**g**) and Th17 to Treg (**h**). Green line: the normal lower limit. Red line: the normal upper limit. Each dot represents single cell. The red numbers in the figure caption represent the percentage of cells in the corresponding quadrants. Bar graphs show the mean ± s.d. * *p* < 0.05; ** *p* < 0.01. NS: no statistical significance.

Next, we analyzed the percentage of some subsets of CD4^+^ T lymphocytes in the asymptomatic and symptomatic COVID-19 individuals. The results showed no differences in the percentage of Th1 in CD4^+^ T lymphocytes, which was in the normal range in both two groups (Figure 3a,c). Additionally, symptomatic individuals had a relatively lower Th2 percentage (beyond the normal range) (Figure 3a,d), a higher Th17 percentage (beyond the normal range) (Figure 3a,c) and a lower Treg percentage (near the lower limit of normal range) (Figure 3b,f). In contrast, asymptomatic cases had a relatively higher Th2 percentage (beyond the normal range) (Figure 3a,d), a relatively lower Th17 percentage (beyond the normal range) (Figure 3a,c) and a higher Treg percentage (in the normal range) (Figure 3b,f).

Those changes led to an abnormally low ratio of Th1 to Th2 (near the lower limit of normal range) (Figure 3g) and high ratio of Th17 to Treg (beyond the normal range) (Figure 3h) in both two groups, suggesting that virus infection can lead to immune imbalance regardless of clinical symptoms. However, asymptomatic cases tend to establish a relatively balanced status by decreasing the percentage of Th17 and increasing the percentage of Treg, and therefore had a relatively lower Th17/Treg ratio (Figure 3h). Additionally, it seemed that the Th1/Th2 ratio of asymptomatic cases was slightly lower, but the differences between the two groups had no statistical significance (Figure 3g).

## 3. Discussion

A vast proportion of asymptomatic COVID-19 individuals may cause an exponential increase in the number of COVID-19 cases globally. As we know, individual immune responses potentially orchestrate the pathology and play important roles in determining different clinical courses of SARS-CoV-2 infection. A recent study showed an extensive and durable remodeling of immune cells during and after SARS-CoV-2 infection [11]. However, the immune mechanisms that are associated with asymptomatic infection are still largely elusive. To reveal possible mechanisms by which a large fraction of COVID-19 patients remain asymptomatic, we aimed to investigate whether the clinical characteristics and immunophenotyping of innate and adaptive immune cells in asymptomatic and symptomatic COVID-19 patients can help us identify pathological or protective immune responses during COVID-19 infection.

Firstly, we tried to find the demographic characteristics and laboratory indicators of asymptomatic individuals based on comprehensive clinical data. Asymptomatic individuals seemed to be younger than symptomatic ones (27.4 ± 9.6 versus 44.9 ± 18.4 years). We found a higher rate of asymptomatic infection in the younger group through age-stratified analysis and a higher rate of symptomatic infection in the older group, which is consistent with the results of Davies et al. [12]. Additionally, the group with chronic diseases had a higher rate of symptomatic infection but a lower rate of asymptomatic infection. This result implied that chronic diseases, such as hypertension and cardiovascular disease, may promote the risk of COVID-19. Many other studies also reported this phenomenon [13,14], which may explain to some extent why those asymptomatic individuals have a shorter hospital stay (16.8 ± 5.2 versus 21.9 ± 8.4 days) and better prognosis. However, there was no significant difference in the re-positive rate between the two groups (20.8% versus 23.5% in asymptomatic and symptomatic individuals, respectively).

In addition, we focused on some parameters reflecting inflammation. The mean value of CRP far exceeded the normal upper limit in symptomatic cases, while it was in the normal range in asymptomatic individuals. Besides, although the value of D-dimer and fibrinogen were in the normal range in the two groups, they were higher in the symptomatic group. Our results implied that the systemic inflammatory response was relatively strong in symptomatic individuals. Consistently, Zhao and colleagues reported that CRP, IL6, LDH and ferritin increased dramatically in COVID-19 patients, leading to disease progression [15].

SARS-CoV-2-infected epithelial cells could recruit and activate various innate immune cells, including macrophages, NK, neutrophils, and others, and adaptive immune cells to orchestrate antiviral immunity and immunopathological effect closely associated with disease severity and progression [16,17]. NK cells play an important role in early immune defense against viral infection, which can lyse virus-infected cells at an early stage of infection and prevent viral dissemination [18]. The decline in the number and function of NK cells likely allows viruses to proliferate intracellularly to trigger NLRP3 activation and aggravate inflammation. Among several pathogenesis mechanisms of COVID-19, hypercytokinemia has taken center stage [19,20]. Recruited monocytes could cause an “atypical” cytokine storm [21], which is characterized by reduced type II interferon signaling, and finally cause local or systemic damage [22]. The balance between monocytes and NK cells may be closely related to the clinical outcome of COVID-19. The results of flow cytometry showed that NK percentage was beyond the normal range and the monocytes percentage was lower than that of healthy control in asymptomatic cases, which could lead to the decrease in the monocytes to NK cells ratio (MNKR) in asymptomatic cases. Similar to our results, Rita et al. also demonstrated the loss of NK cells and the expansion of monocytes in mild or severe COVID-19 [23]. However, Diao et al. and Zheng et al. observed NK cell cytopenia in severe COVID-19 patients [24,25], which was contrary to our results. These results suggest that the change pattern of NK and monocytes may be one of the sensitive and important factors affecting the prognosis of the disease, which still needs to be further clarified.

Additionally, the progression of the disease is not only related to the frequency of NK and monocytes, but also to their phenotypes. A single cell sequencing study identified 7 monocyte clusters and 13 major NK cell clusters in PBMC from COVID-19 patients, respectively. Different clusters had different transcriptional characteristics and functional phenotypes. The proportion of different subpopulations was closely related with severity of COVID-19 [11]. In the future, to accurately reflect the characteristics of immune phenotype of asymptomatic and symptomatic cases, it is necessary to analyze NK and monocyte clusters.

We comprehensively analyzed the number and percentage of various peripheral blood leukocytes (including lymphocytes, granulocytes and monocytes) of asymptomatic and symptomatic SARS-CoV-2-infected people. Previous studies have shown that most COVID-19 patients have lymphopenia, and the degree of lymphocyte reduction is closely related to the severity of COVID-19 [26,27]. It has been shown that the number of neutrophils and monocytes increased abnormally with the decrease in the number of lymphocytes in patients with COVID-19 [28,29], which can lead to the increase in the neutrophil to lymphocyte ratio (NLR) and monocyte to lymphocyte ratio (MLR), especially in patients with severe COVID-19. Our results showed the differences of changes in lymphocytes and monocytes between symptomatic and asymptomatic cases. Symptomatic cases had an abnormally low lymphocyte count and percentage, and a normal monocyte percentage. In contrast, asymptomatic cases had a normal level of lymphocyte and a low percentage of monocyte, which was near the normal lower limit. This immune phenotype of asymptomatic cases cannot only help patients maintain a protective immune response, but also avoid inflammatory injury mediated by monocytes, which may be one of the reasons for their asymptomatic state.

As an intracellular parasitic microorganism, cellular immunity plays an important role in antiviral immunity. Besides, cellular immunity can regulate innate immune and humoral immune response [30]. Therefore, it is important to investigate cellular immune function to understand the immune mechanisms of asymptomatic infection and disease progression. This study also focused on the changes in CD4^+^ T cell subsets, including Th1, Th2, Th17, and Treg, which play different roles in the antiviral immunity. Th1 subsets play an important role in activating macrophage-mediated inflammation and cell-mediated immunity [31]. In addition to mediating humoral immune response, the Th2 population can inhibit inflammation, which Th1 and Th17 trigger by regulating IFN-mediated response [32]. Th17 mediates antiviral inflammation by secreting IL17. Some studies have shown that Th17 and IL17 increased significantly in patients with COVID-19, which participate in cytokine storm and disease progression. Th17/IL17 axis may be used as a clinical therapeutic target for COVID-19 [33]. Treg could suppress the immune response and maintain immune balance by secreting anti-inflammatory cytokines such as TGFβ, IL10 and IL35. Th1/Th2 and Th17/Treg balance play crucial roles in various diseases, such as autoimmune diseases and viral infections. The imbalance of the immune system can increase various inflammatory cytokines such as MIP-1, GM-CSF, and IL1β, leading to respiratory injury and accelerating COVID-19 [34]. Do asymptomatic individuals establish their asymptomatic carrier state by inducing T cell response towards an anti-inflammatory phenotype and maintaining immune balance?

We investigated the changes in Th1, Th2, Th17, Treg, Th1/Th2 ratio, Th17/Treg ratio in asymptomatic and symptomatic patients with COVID-19. Our results showed that symptomatic and asymptomatic individuals both had an abnormally high percentage of Th2 and Th17, which can lead to the abnormally low ratio of Th1 to Th2 and high ratio of Th17 to Treg, suggesting that virus infection can lead to immune imbalance regardless of clinical symptoms. Compared to the symptomatic cases, asymptomatic individuals had a relatively lower Th17 percentage and a higher Treg percentage, which suggested that asymptomatic cases tend to establish a relatively balanced immune phenotype by decreasing Th17/Treg ratio. A study of RNA sequencing recently explored differentially expressed genes of symptomatic and asymptomatic patients with COVID-19. The results showed a higher expression of the genes involved in the Th2 pathway, together with the down-regulation of the genes in Th1 and Th17 pathways in asymptomatic individuals [35]. Our results are consistent with theirs. Both high-throughput data and our results suggest that immune homeostasis plays a crucial role in COVID-19 progression. Asymptomatic individuals may be prone to establishing a relatively balanced immune response by decreasing Th17 populations and increasing Treg populations, which can regulate the excessive increase in inflammatory responses of COVID-19 patients.

In the past year, multiple studies were conducted to evaluate the role of CD4 T cell subsets in the pathogenesis of COVID-19. However, these studies report conflicting results. Some studies showed that Th1 subsets and their cytokines increased significantly in patients with COVID-19 [36]. In contrast, some studies have yielded opposite results [37,38]. However, in our investigation, the percentage of Th1 in asymptomatic individuals is equal to that in symptomatic patients. Several studies showed that the levels of Th2 subsets and their cytokines in COVID-19 patients were significantly increased, especially in severe COVID-19 patients [39]. In contrast, another study found that theTh2 population and their cytokines were decreased in hospitalized patients compared to asymptomatic individuals [28], which is consistent with our results. Most studies reported that Th17 subsets and their cytokines were increased, while the proportion of Treg was decreased in patients with COVID-19 [40,41,42,43], which may lead to cytokine storm and promote disease progression. However, another piece of research observed a significant increase in the number of Treg cells in patients with COVID-19. As discussed above, various studies have conflicting results. The possible reasons are as follows: firstly, the subjects included in different studies have a diverse demographic and genetic background and disease severity, but they are not divided into different groups according to the severity of the disease. Secondly, the type of detection techniques used in different studies may affect the results. Thirdly, the peripheral blood sample may be collected before or after clinical treatment, influencing the results.

Antiviral drugs used in this study, such as lopinavir or interferon, have immunomodulatory effects. Lopinavir can inhibit the production of TNFα and other pro-inflammatory cytokines in human endothelial cells, inhibit T lymphocytes proliferation and reactivity, and exhibit an inhibitory effect on proteasome activities to reduce transcription of genes encoding pro-inflammatory cytokines [44,45,46]. Surely it can improve inflammatory status in COVID-19 patients and be a confounder for our results. Type Ⅰ interferon is acknowledged for its antiviral roles. Additionally, interferon may confer immunopathological effects by inducing excessive inflammation in viral infection. IFN can trigger the expression of a diverse suite of IFN-stimulated genes (ISGs), including a large number of proinflammatory cytokines and chemokines, such as CXCL10, MCP1, MIP1β, and IL12. These chemokines can recruit monocytes and CXCR3 expressing Th1 cells producing many proinflammatory cytokines and chemokines, which finally skewed the host immune response [47]. Therefore, lopinavir or interferon may differentially regulate inflammatory mediators of COVID-19. To avoid the impact of medical treatment in our results, we collect blood samples before any clinical intervention. In addition, small sample size may increase the bias. In our study, some indicators, such as the NK percentage, were higher in asymptomatic cases but without statistical significance. The possible reason is that the number of samples is too small to result in negative results, which is certainly a limitation of our study. Therefore, more studies need to be conducted in the future.

Overall, symptomatic individuals were prone to establishing a non-protective immune phenotype by abnormally decreasing the lymphocyte count and percentage, abnormally increasing the Th17 percentage and decreasing Treg percentage. In contrast, asymptomatic individuals tended to establish a more effective and protective immune phenotype by maintaining a normal level of lymphocyte and a high level of NK cells. At the same time, asymptomatic individuals can establish a relatively balanced immune response through maintaining a low level of monocytes, a relatively low level of Th17 and high level of Treg, which therefore lead to the decrease in MNKR and Th17/Treg ratio and finally avoidance of excessive inflammatory responses. This may be one of the reasons for their asymptomatic states. This study is helpful to reveal the immunological characteristics of asymptomatic individuals, understand immune pathogenesis of COVID-19 and predict clinical outcomes more precisely. However, owing to small sample sizes, a future study with larger sample size is still warranted.

## 4. Materials and Methods

### 4.1. Study Design and Participants

The study group enrolled patients with COVID-19 (aged 17–67 years) hospitalized in the Fifth Hospital of Shijiazhuang from January to June 2020. A total of 41 COVID-19-infected individuals were included for flow cytometry detection in this study (including 24 asymptomatic individuals and 17 symptomatic cases). For immunological indexes with normal reference values, we did not set a healthy control, and compared the results with the normal reference values. For immunological indicator without normal reference values, such as the percentage of monocyte in karyocyte, we detected 20 healthy individuals who participated in physical examination in the Fifth Hospital of the Shijia Zhuang at the same time to be as the baseline. For the blood routine examination, to better observe the changing trend, we collected the blood routine results of 50 age-matched healthy controls in the same period to be the baseline.

The symptomatic COVID-19 infections were defined as follows: positive test results for SARS-CoV-2 by qPCR in oro/nasopharyngeal swab samples plus being symptomatic. The asymptomatic infection was defined as follows: positive test for SARS-CoV-2 by qPCR but no symptoms throughout the 14-day quarantine according to the standard definition in China, which is different from that of the World Health Organization. According to the management standards for an asymptomatic infected person launched by The Joint Prevention and Control Mechanism of the State Council of China, there are two types of asymptomatic carriers: those who are medically quarantined for 14 days without any self-perceiving or clinically identifiable symptoms, and those who are in the incubation period of symptomatic infection. The second type of asymptomatic carrier may become a symptomatic patient during quarantine. In our study, only the first type of asymptomatic case was included.

Asymptomatic individuals are at risk of spreading the virus. In order to better control the epidemic of COVID-19, the Chinese government has adopted very strict centralized isolation of fixed medical organization for suspected cases, confirmed cases and asymptomatic cases. According to Prevention and Control of COVID-19 (Tentative 4th Edition) of the National Health Commission of the People’s Republic of China, 14 days of centralized isolation and medical observation shall be carried out for asymptomatic cases identified by medical care and health institutions at various levels and of different types. Additionally, to determine different clinical types of confirmed COVID-19 and predict the progression of asymptomatic cases precisely, CT chest images are carried out routinely for all cases. These strict isolation measures for asymptomatic individuals conducted in China may be different from other countries.

All patients received comprehensive clinical examination. The following variables were collected for each patient: age, sex, complication, epidemiological data, past or present medical history, clinical manifestations, severity assessment on admission, laboratory findings, the results of chest computed tomography (CT) scan, treatment, hospitalization days and clinical outcome, etc. Additionally, peripheral blood was collected to detect the number and percentage of various immune cells and their subsets by flow cytometry.

### 4.2. Flow Cytometry

Venous blood samples were collected from the patients on hospital admission and transferred to the laboratory within 1 h. Immunophenotyping of immune cells and lymphocyte subsets was performed using a 3-laser, 10-color FACSCanto II flow cytometer (Beckman-Coulter, Brea, CA, USA). All ethylenediaminetetraacetic acid (EDTA)-anticoagulated blood samples were analyzed immediately as soon as the blood was collected. Sample preparation and analysis were performed according to the manufacturer’s instructions. About 100 µL aliquots of blood were incubated with 2 mL of BD FACS^TM^ Lysing Solution (Cat No. 349202, BD Biosciences, Franklin Lakes, NJ, USA) in the dark for 10 min to lyse the red blood cells. After washing and re-suspending the cells in 100 µL PBS, the cells were vortexed and incubated in the dark with combinations of the following fluorochrome-conjugated antibodies for 30 min at 4 °C. CD16/32 monoclonal antibody was pre-incubated with the cells at room temperature for 10 min to block FcR of B cells and monocytes. Finally, stained samples were analyzed with FACSCanto II flow cytometer immediately after completion of sample staining. Data analyses were performed with the BD FACS Diva software. The antibodies used in this study are as follows: CD56-BB700 (566573, BD), CD45-APC (555485, BD), CD8-PE-Cy7 (557746, BD), CD4-PE-Cy7 (557852, BD), CD16-APC-H7 (560195, BD), CD45-Percp-Cy5.5 (564105, BD), CD196-APC (560619, BD), CD4-PE-Cy7 (560909, BD), CD4-PE (555347, BD), CD19-APC (555415, BD), CD25-PE (555415, BD), CD86-PE (555658, BD), CD3-FITC (555916, BD), CD183-PE (557185, BD), CD127-Alexa Fluor^®^ 647 (558598, BD), CD14-PE-Cy7 (557742, BD), CD16/CD32 antibody (553141, BD), BD FACS lysing solution (349202, BD).

The gating strategy used in the study for immune cells identification is shown in Figure S1. Firstly, after the standard rejection of debris, residual erythrocytes and doublets, physical gating was performed based on CD45 and side scatter (SSC) to distinguish the lymphocytes group, monocytes, and granulocytes. We gated lymphocytes as CD45 ^bright^/SSC ^low^ cells, monocytes as CD45 ^bright^/SSC ^mid^ cells, and granulocytes as CD45^+^/SSC ^high^ cells. In the second step, the following subpopulations of monocytes and lymphocytes were defined and gated as follows: B cells (CD3^−^CD19^+^); CD4^+^T cells (CD3^+^ CD4^+^); CD8^+^T cells (CD3^+^CD8^+^); Th1 lymphocyte (CD3^+^CD4^+^CD183^+^CD196^−^); Th2 lymphocyte (CD3^+^ CD4^+^ CD183^−^CD196^−^); Th17 lymphocyte (CD3^+^CD4^+^CD183^−^ CD196^+^); Treg (CD3^+^CD4^+^CD25^+^CD127^low^); NK cell (CD3^−^CD56^+^); monocyte cells (CD3^−^CD14^+^).

### 4.3. RNA Extraction and Quantitative Real-Time PCR (qPCR) to Detect SARS-CoV-2 RNA

Clinical samples (nasopharyngeal swabs/pharyngeal swabs/anal swab) were collected according to the Technical Guidelines for COVID-19 Laboratory Testing formulated by the National Health Commission of China [48]. Viral RNA extraction and nucleic acid detection were performed within 24 h of collecting the samples. DNA and RNA extraction kits (Zybio, Chongqing, China) were used to extract SARS-CoV-2 RNA according to the manufacturer’s instructions. RNA was extracted from 200 µL of samples, before being eluted in 50 µL of elution buffer and used as the template for qPCR. SARS-CoV-2 RNA was detected using novel coronavirus nucleic acid detection kit (DA AN Gene, Guangzhou, China) according to the manufacturer’s protocol. Briefly, 5 µL RNA samples were added into 17 µL PCR Reaction Mix A and 3 µL PCR Reaction Mix B and analyzed using ABI prism 7500 Sequence Detection System (Applied Biosystems, Waltham, MA, USA). Amplification was performed as follows: 50 °C for 2 min, 95 °C for 2 min, followed by 42 cycles consisting of 95 °C for 5 s, 60 °C for 35 s and a default melting curve step in an ABI 7500 machine. At the same time each PCR run included positive and negative control tubes.

### 4.4. Statistical Analysis

All data were statistically analyzed by SPSS20.0 software (SPSS lnc., IBM Corporation, Chicago, IL, USA). The measurement data were expressed by mean ± standard deviation (s.d.). Data were checked for normality and homogeneity of variances. For normally distributed data, an unpaired Student’s t-test was applied for statistical analysis between the two groups, and one-way analysis of variance (ANOVA) was used for multiple groups. Non-parametric tests were used when data were not normally distributed. Mann–Whitney U test and Kruskal–Wallis H test were applied to compare the differences between the two groups and among multiple groups, respectively. The count data were expressed in the form of a percentage, and the Pearson chi-square test or Fisher’s precision probability test was used for comparing the groups. Graph Prism software was utilized to plot the graphs. A value of *p* ≤ 0.05 was considered to be significant statistically.

## Figures and Tables

**Table 1:** The demographic characteristics of symptomatic and asymptomatic cases.

Characteristics	Symptomatic Cases (*n* = 17)	Asymptomatic Cases (*n* = 24)
**Age (years)**		
Mean ± S.D.	44.9 ± 18.4	27.4 ± 9.6
Median	44	23.5
**Sex**		
Male	64.7% (11)	41.7% (10)
Female	35.3% (6)	58.3% (14)
**Smoking habit**		
Non-smokers	76.5% (13)	95.8% (23)
Smokers	23.5% (4)	4.2% (1)
**Source**		
Mainland	47.1% (8)	29.2% (7)
Overseas	52.9% (9)	70.8% (17)
**Been to the epidemic area**		
Yes	29.4% (5)	41.7% (10)
No	70.6% (12)	58.3% (14)
**Exposure to confirmed cases**		
Yes	29.4% (5)	33.3% (8)
No	70.6% (12)	41.7% (16)

**Table 2:** The effect of age on the rates of symptomatic and asymptomatic infection.

Age	Asymptomatic Case (*n* = 24)	Symptomatic Cases (*n* = 17)	χ^2^	*p*
The Older group (≤40 years)	2 (15.4%)	11 (84.6%)	14.604	<0.01
The younger group (>40 years)	22 (78.6%)	6 (21.4%)		

**Table 3:** The effect of gender on the rates of symptomatic and asymptomatic infection.

Gender	Asymptomatic Case (*n* = 24)	Symptomatic Cases (*n* = 17)	χ^2^	*p*
Male	10 (47.6%)	11 (52.4%)	2.114	>0.05
Female	14 (70%)	6 (30%)		

**Table 4:** The effect of smoking on the rates of symptomatic and asymptomatic infection.

Smoking Habits	Asymptomatic Case (*n* = 24)	Symptomatic Cases (*n* = 17)	χ^2^	*p*
Smokers	1 (20%)	4 (80%)	3.484	>0.05
Non-smokers	23 (63.9%)	13 (36.1%)		

**Table 5:** The comparison of the rates of symptomatic and asymptomatic infection between local and overseas cases.

Case Source	Asymptomatic Cases (*n* = 24)	Symptomatic Cases (*n* = 17)	χ^2^	*p*
Local cases	7 (46.7%)	8 (53.3%)	1.373	>0.05
Overseas cases	17 (65.4%)	9 (34.6%)		

**Table 6:** The clinical characteristics of symptomatic and asymptomatic cases.

Clinical Characteristics	Symptomatic Cases (*n* = 17)	Asymptomatic Cases (*n* = 24)
**Symptoms**		
Fever	41.2% (7)	0
Headache	17.6% (3)	0
Cough	17.6% (3)	0
Pharyngalgia	5.9% (1)	0
Runny Nose	11.8% (2)	0
Digestive tract symptoms	17.6% (3)	0
**Time to diagnosis (days)**	6.1 ± 8.1	2.0 ± 0.6
**Nucleic acid testing**		
Throat swab	82.4% (14)	62.5% (15)
Nose swab	47.1% (8)	50.0% (12)
Anal swab	29.4% (5)	37.5% (9)
**Coronavirus antibodies**		
IgG	71.4% (5/7)	84.2% (16/19)
IgM	14.3% (1/7)	21.1%(4/19)
**complications**		
Hypertension	17.6% (3)	4.2% (1)
Cardiovascular disease	17.6% (3)	4.2% (1)
Respiratory disease	5.9% (1)	0
Cancer	0	0
Diabetes	0	0
Kidney disease	5.9% (1)	0
The history of blood transfusion	11.8% (2)	0
Allergic history	5.9% (1)	8.3% (2)
**Disease classification**		
Asymptomatic infection	0% (0)	100% (24)
Mild	88.2% (15)	0% (0)
Severe	11.8% (2)	0% (0)
**Lung CT**		
Inflammation	70.6% (12)	20.8% (5)
Pneumatocele	11.8% (2)	4.2% (1)
Other (nodule, cord, pleural thickening, pleural thickening)	35.3% (6)	12.5% (3)
**Length of stay**		
Mean ± S.D.	21.9 ± 8.4	16.8 ± 5.2
**Treatment**		
**Antibiotic**	0	12.5% (3)
**Antiviral**		
Arbidol	41.7% (10)	95.8% (23)
Traditional Chinese medicine	76.5% (13)	95.8% (23)
Lopinavir/ Ritonavir	35.3% (6)	0
Human Immunoglobulin (I.V)	0	0
Glucocorticoid	0	0
Hydroxychloroquine	23.5% (4)	4.2% (1)
IFN-α (INH)	94.1% (16)	100% (24)
**Oxygen intracavitary**		
low-flow nasal oxygen	23.5% (4)	0
Noninvasive mechanical ventilation	0	0
Invasive mechanical ventilation	0	0
**Clinical outcome**		
Death	0	0
Re-positive	23.5% (4)	20.8% (5)

**Table 7:** The effect of chronic diseases on the rates of symptomatic and asymptomatic infection.

Complications	Asymptomatic Cases (*n* = 24)	Symptomatic Cases (*n* = 17)	χ^2^	*p*
With chronic diseases	2 (20%)	8 (80%)	8.092	<0.01
Without chronic diseases	22 (71.0%)	9 (29%)		

**Table 8:** The comparison of the re-positive rate between symptomatic and asymptomatic group.

The type of Infection	Non-Re-Positives	Re-Positives	χ^2^	*p*
Asymptomatic cases (*n* = 24)	19 (79.2%)	5 (20.8%)	0.042	>0.05
Symptomatic cases (*n* = 17)	13 (76.5%)	4 (23.5%)		

## Data Availability

The data presented in this study are available on request from the corresponding author.

## Supplementary Materials

The following are available online at https://www.mdpi.com/article/10.3390/pathogens10091105/s1, Figure S1: The age (a) and hospital days (b) were compared between asymptomatic and symptomatic COVID-19 individuals. Bar graphs show the means ± s. d. * *p* < 0.05; *** *p* < 0.001. Figure S2: The count and the percentage of various cells in peripheral blood in healthy control, asymptomatic and symptomatic individuals with COVID-19. Peripheral blood was collected immediately after the cases were hospitalized, and the following indexes, including red blood cell count (a), white blood cell count (b), neutrophil count and percentage (c), lymphocyte count and percentage (d), monocyte count and percentage (e), the ratio of neutrophil/lymphocyte (NLR) (f) and the ratio of monocyte/lymphocyte (MLR) (g) were analyzed between three groups. Green line: the normal lower limit. Red line: the normal upper limit. Bar graphs show the means ± s. d. * *p* < 0.05; ** *p* < 0.01; *** *p* < 0.001. NS: no statistical significance. Figure S3: The comparison of various blood coagulation indexes and acute phase reactants between asymptomatic and symptomatic patients with COVID-19. Peripheral blood was collected immediately after the cases were hospitalized, and the following blood coagulation indexes, including D-Dimer (a), fibrinogen (b), platelet count (c), prothrombin time (d), activated partial thromboplastin time (e), international normalized ratio (f) and C-reactive protein (CRP) (g) were analyzed between asymptomatic and symptomatic cases. Green line: the normal lower limit. Red line: the normal upper limit. Bar graphs show the means ± s.d. * *p* < 0.05, *** *p* < 0.001. NS: no statistical significance. Figure S4: Gating strategy used in the study for immune cells identification.
