# Supplementary material for: Asymptomatic COVID-19 Individuals Tend to Establish Relatively Balanced Innate and Adaptive Immune Responses
Source: Pathogens. 2021 Aug 30;10(9):1105. doi: 10.3390/pathogens10091105 (PMC8468997; doi:10.3390/pathogens10091105)
Supplement: Supplementary file 1 [file pathogens-10-01105-s001.zip › pathogens-1300940-supplementary.pdf]

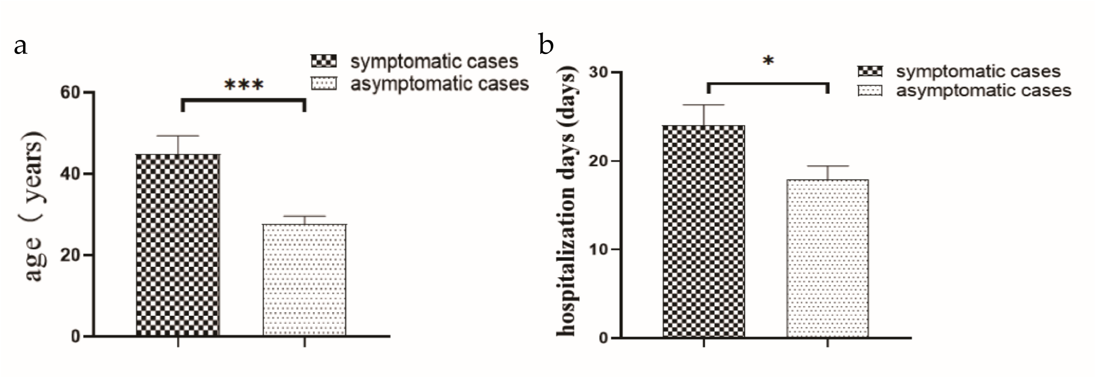

Figure S1. The age (a) and hospital days (b) were compared between asymptomatic and symptomatic COVID-19 individuals. Bar graphs show the means ± s. d. * P<0.05; ***P<0.001.

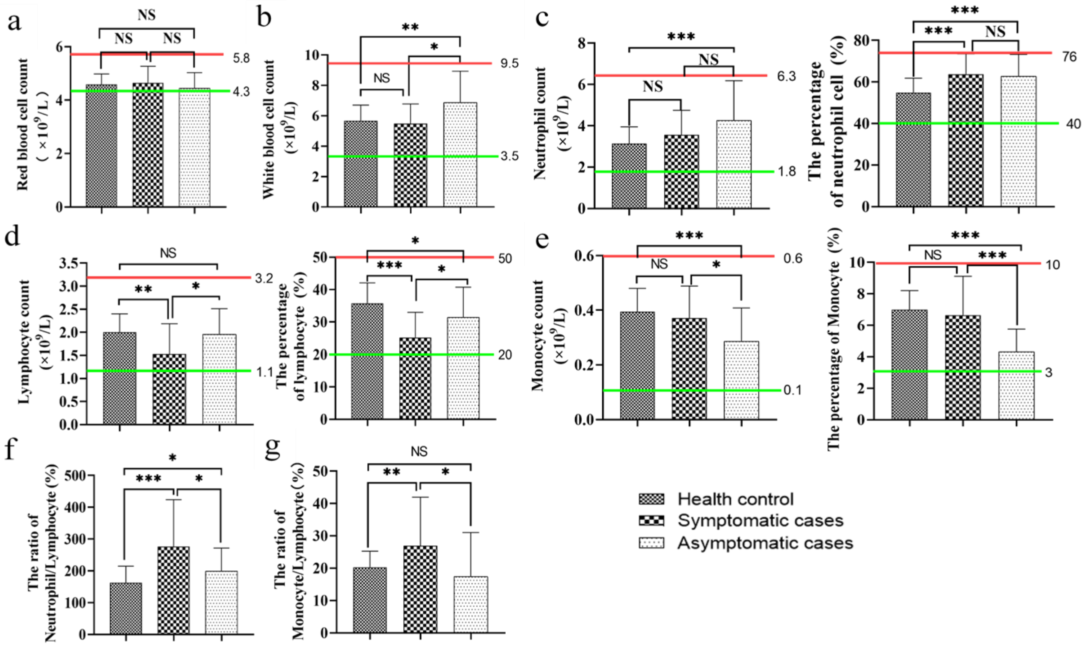

Figure S2. The count and the percentage of various cells in peripheral blood in healthy control, asymptomatic and symptomatic individuals with COVID-19. Peripheral blood was collected immediately after the cases were hospitalized, and the following indexes, including red blood cell count (a), white blood cell count (b), neutrophil count and percentage (c), lymphocyte count and percentage (d), monocyte count and percentage (e), the ratio of neutrophil/lymphocyte (NLR) (f) and the ratio of monocyte/lymphocyte (MLR) (g) were analyzed between three groups. Green line: the normal lower limit. Red line: the normal upper limit. Bar graphs show the means ± s. d. * P<0.05; ** P<0.01; ***P<0.001. NS: no statistical significance.

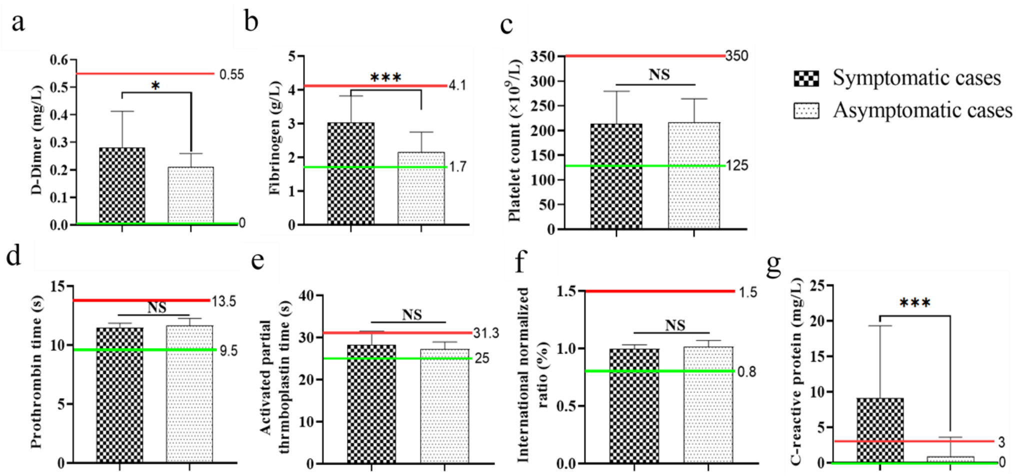

Figure S3. The comparison of various blood coagulation indexes and acute phase reactants between asymptomatic and symptomatic patients with COVID-19. Peripheral blood was collected immediately after the cases were hospitalized, and the following blood coagulation indexes, including D-Dimer (a), fibrinogen (b), platelet count (c), prothrombin time (d), activated partial thromboplastin time (e), international normalized ratio (f) and C-reactive protein (CRP) (g) were analyzed between asymptomatic and symptomatic cases. Green line: the normal lower limit. Red line: the normal upper limit. Bar graphs show the means ± s.d. * *P*<0.05, ****P*<0.001.. NS: no statistical significance.

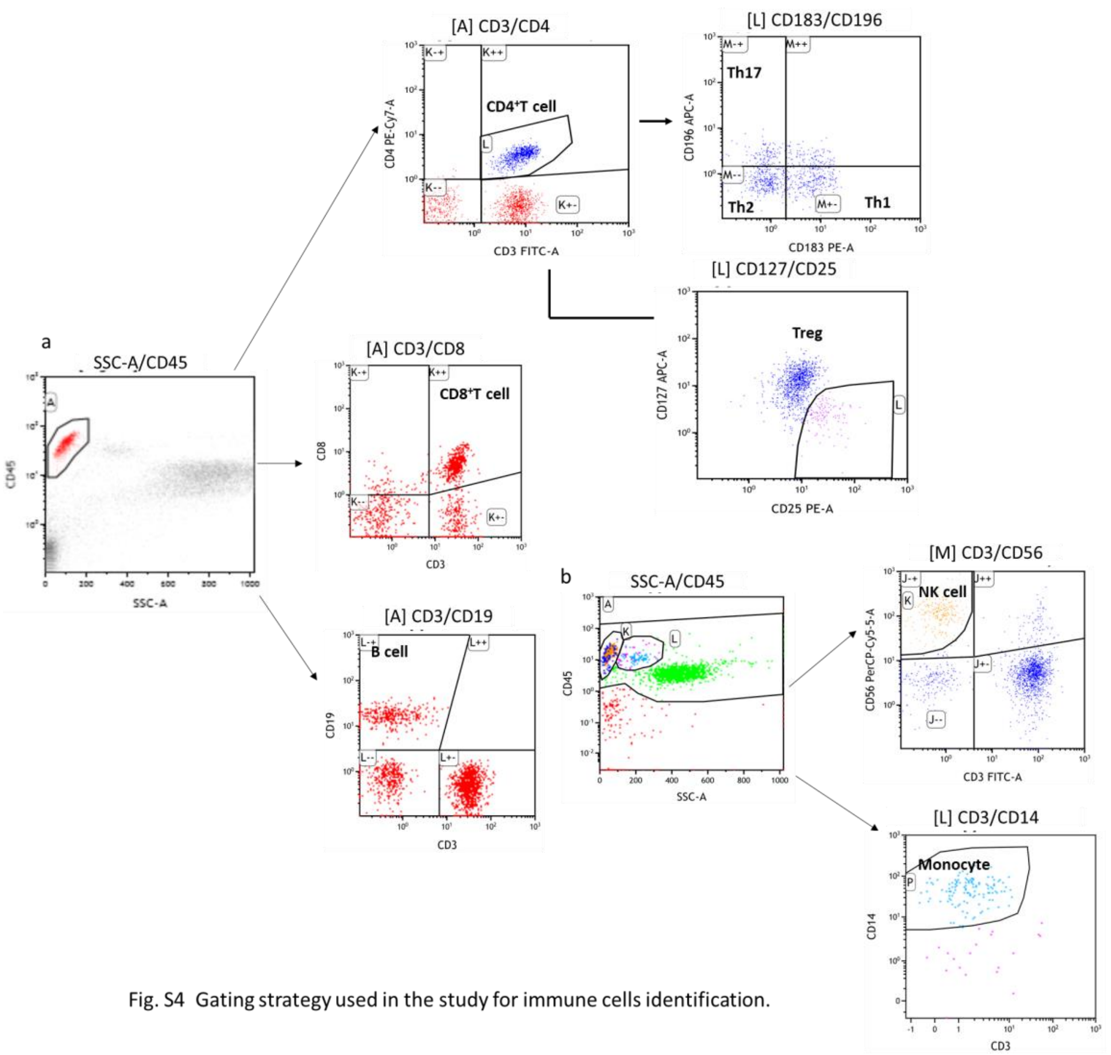

Figure S4. Gating strategy used in the study for immune cells identification.
